# Large Interstitial Ectopic Pregnancy: Management by Laparoscopic Cornuostomy Following Initial Misdiagnosis

**DOI:** 10.7759/cureus.19280

**Published:** 2021-11-05

**Authors:** Shweta Mittal, Bhawani Shekhar

**Affiliations:** 1 Centre of IVF and Human Reproduction, Department of Obstetrics and Gynaecology, Sir Gangaram Hospital, New Delhi, IND

**Keywords:** cornuostomy, laparoscopy, ultrasonography, missed diagnosis, interstitial pregnancy

## Abstract

The purpose of the present report is to highlight the challenges in diagnosing interstitial ectopic pregnancy and to describe its management by laparoscopic cornuostomy. A 28-year-old gravida 3, para 1 woman was referred to us at 12 weeks period of gestation after failed medical termination following a diagnosis of missed abortion. On presenting to us, a large interstitial ectopic pregnancy was diagnosed by ultrasonography and managed by laparoscopic cornuostomy. Intra myometrial vasopressin and purse string sutures at the base of ectopic pregnancy bulge were used to reduce intra-operative bleeding. Intra-operative blood loss was 50 ml. Patient was discharged after two days of surgery. Interstitial pregnancy may be misdiagnosed as an intrauterine pregnancy, due to lack of suspicion and expertise. Large interstitial ectopic pregnancies can be successfully managed by a conservative surgical approach such as laparoscopic cornuostomy instead of cornual resection or hysterectomy.

## Introduction

Interstitial ectopic pregnancy is defined as a gestation which implants within the proximal tubal segment that lies within the muscular uterine wall [1]. Interstitial pregnancies are rare, accounting for 2-4% of all ectopic pregnancies [2]. They are associated with high risk of uncontrolled hemorrhage and may be life threatening. Maternal mortality rate in interstitial pregnancies is 2-2.5%, which is seven times higher than ectopic pregnancies overall [3]. Interstitial pregnancy requires early diagnosis and prompt management. Despite access to early pregnancy ultrasounds in the current times, it may sometimes be misdiagnosed as an intrauterine pregnancy. Traditionally, surgical treatment of interstitial pregnancy included cornual resection or hysterectomy. Nowadays, a more conservative surgical approach such as cornuostomy is being preferred over cornual resection or hysterectomy and laparoscopy is being preferred over laparotomy [1]. We describe a case of interstitial pregnancy initially misdiagnosed as missed abortion and eventually managed successfully by laparoscopic cornuostomy.

## Case presentation

**Figure 1:**
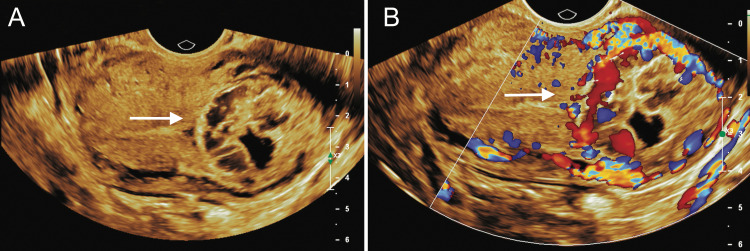
A: 2D Transvaginal ultrasound in transverse view showing a heteroechoic mass in left cornua B: 2D Transvaginal ultrasound in transverse view with colour doppler showing intense vascularity within the cornual mass

**Figure 2:**
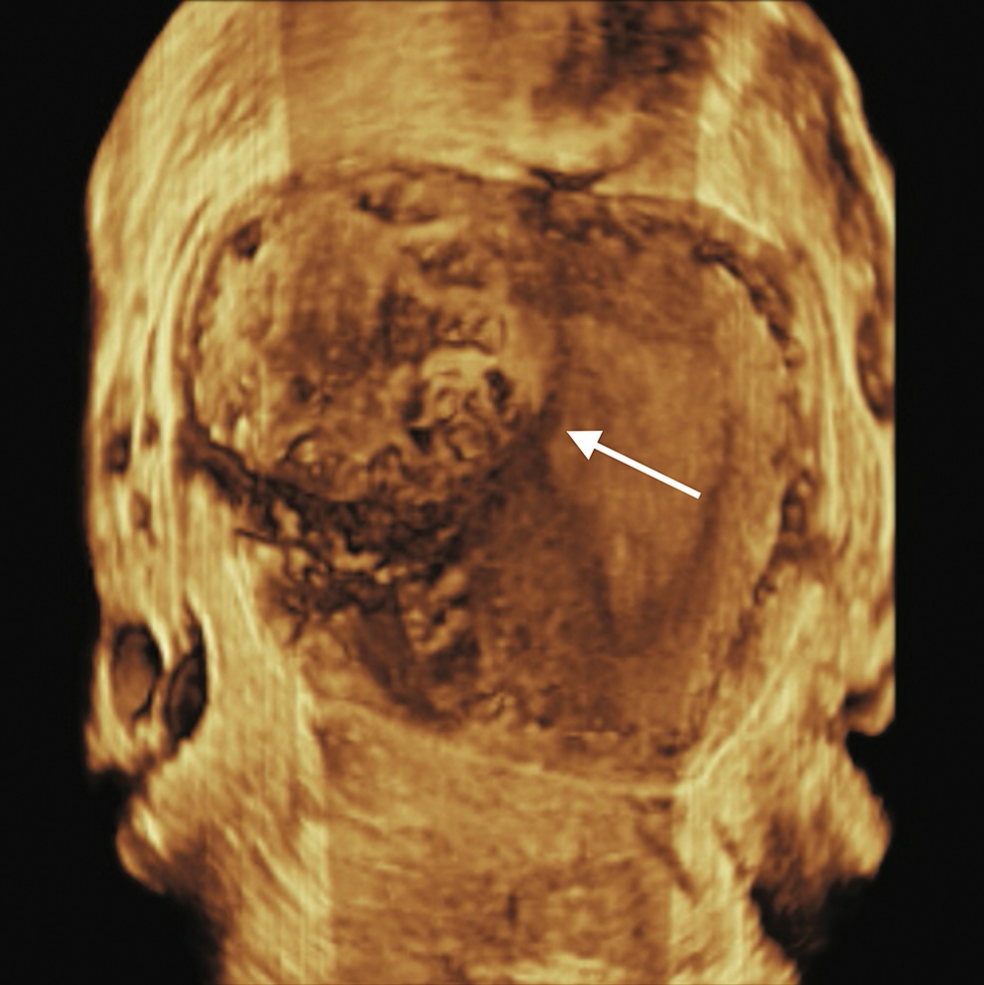
3D Transvaginal ultrasound image showing left cornual mass bulging into serosa

A 28-year-old, naturally conceived third gravida with previous one normal vaginal delivery and one miscarriage presented to a local practitioner at eight weeks gestation with mild vaginal bleeding. Ultrasound was suggestive of an intrauterine non-viable pregnancy corresponding to six weeks gestation near the cornual region. It was diagnosed as a case of missed abortion following which she was prescribed misoprostol for medical termination by a local practitioner. After one week a repeat ultrasound showed similar findings for which she received a second dose of misoprostol. Three weeks later she was referred to our clinic at 12 weeks gestation with ongoing vaginal bleeding and pain abdomen since four days. At presentation, she was hemodynamically stable. Abdominal examination did not reveal any abnormality. On per vaginal examination os was closed with mild bleeding. Uterus was retroverted, parous size with left fornix fullness and tenderness. Haemoglobin was 9.6 g/dl. Serum beta human chorionic gonadotropin (hCG) was 798 mIU/ml. Transvaginal ultrasound (2D and 3D) revealed a 4x3.7x2.7 cm heteroechoic mass with an ill-defined gestational sac within it corresponding to six weeks two days extending from the left cornual edge to the serosa with a bulge on serosal surface showing intense vascularity suggestive of a large interstitial ectopic pregnancy (Figures 1, 2). 

**Figure 3:**
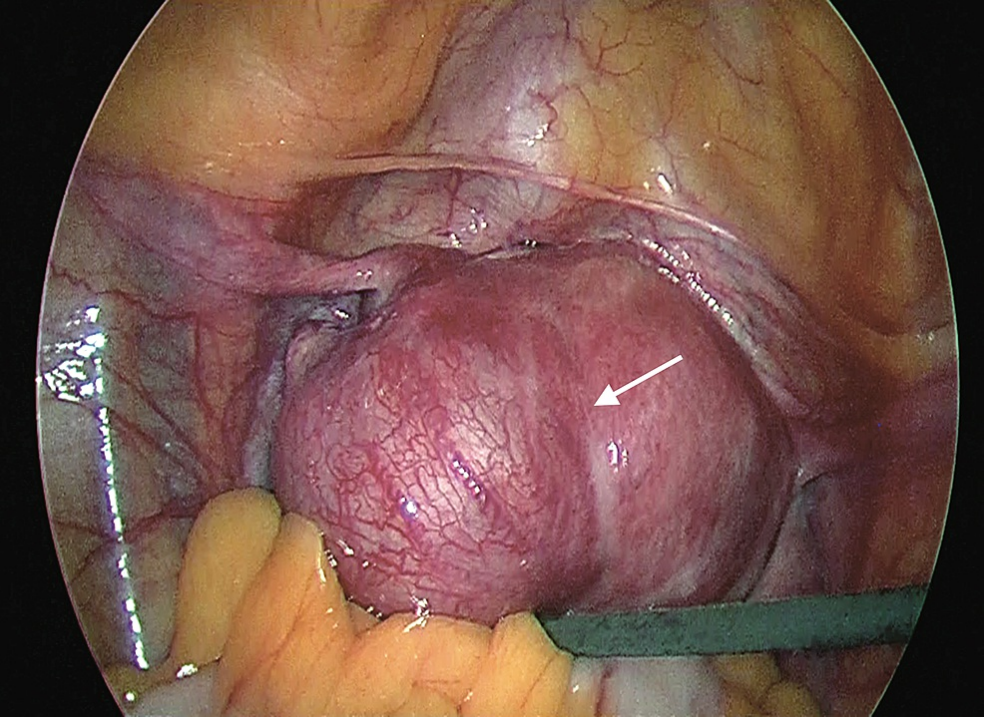
Bulge lateral to the insertion of round ligament confirming interstitial pregnancy

Although serum beta hCG was low, in view of patient’s clinical features and large size of interstitial ectopic pregnancy, surgical management was considered most appropriate. On laparoscopy, a 4x4 cm bulge with increased vascularity was seen in close proximity to the left cornual region of the uterus. Pregnancy bulge was located lateral to the insertion of the round ligament in the uterus, confirming diagnosis of interstitial ectopic pregnancy (Figure 3).

**Figure 4:**
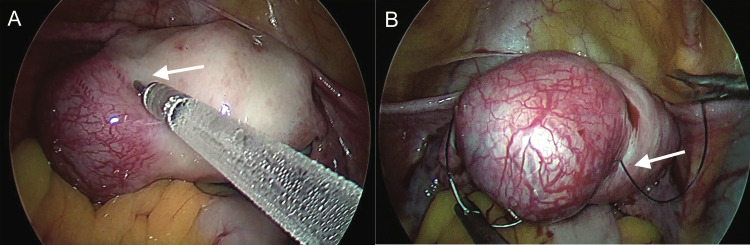
A: Intramyometrial vasopressin injection in peri-cornual area B: Purse string suture encircling cornual bulge

Decision to perform laparoscopic cornuostomy was taken. Prior to incision diluted vasopressin (20 units in 200 ml normal saline) was injected intramyometrially in the peri-cornual area until the myometrium was blanched (Figure 4A). Purse string suture encircling the ectopic pregnancy bulge was applied using 2-0 Vicryl (Figure 4B).

**Figure 5:**
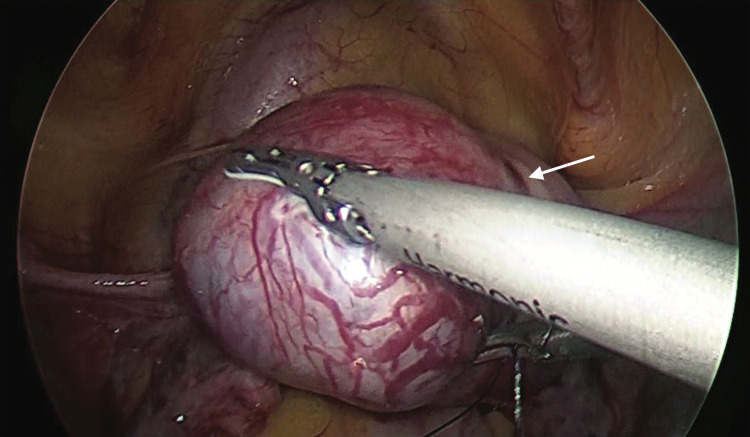
Incision over the cornual bulge with harmonic scalpel

The two ends of the purse string suture were tightly held together with Maryland forceps. This purse string suture helped to reduce the vascularity of the interstitial region and also to stabilize the ectopic pregnancy bulge during surgery. Incision was given over the bulge with harmonic scalpel (Figure 5).

**Figure 6:**
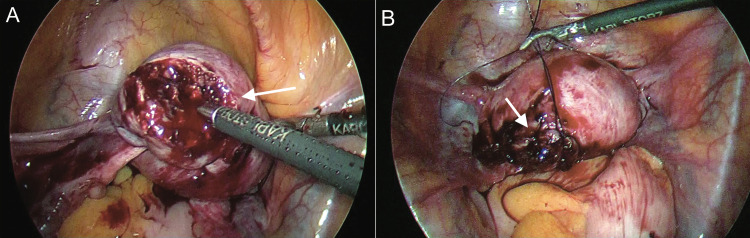
A: Ectopic tissue removed piecemeal with a non tooth grasping forceps B: Uterine wall sutured with 2-0 vicryl suture

Ectopic tissue was removed in a piecemeal fashion in an endobag with the help of non-toothed grasping forceps (Figure 6A). After removal of all ectopic tissue, the uterine wall was sutured in a continuous manner in two layers with 2-0 Vicryl (Figure 6B). 

Purse string suture was removed and haemostasis ensured. Operative time was 60 minutes with 50 ml intra-operative blood loss. The postoperative period was uneventful. Patient was discharged after two days of surgery. Histopathology revealed products of conception. Follow up ultrasound done after two weeks showed no residual ectopic mass. Serial beta hCG monitoring was done weekly till it became <5 mIU/ml three weeks later.

## Discussion

Interstitial ectopic pregnancies are associated with high morbidity and mortality. Excessive bleeding may occur due to rupture, because of the close relation of the ectopic gestation to the intra-myometrial uterine vasculature [2]. They usually present relatively late and are larger in size when they rupture compared to tubal ectopic pregnancies because the layer of overlying myometrium can accommodate larger pregnancies [1].

Interstitial pregnancy may be misdiagnosed as ‘angular’ pregnancy or may be incorrectly termed as ‘cornual’ pregnancy. It is important to differentiate between these three entities, as the associated maternal morbidity and management vary greatly. ‘Angular pregnancy’ is an intra-uterine pregnancy that occurs when the gestational sac is implanted medial to the utero-tubal junction and round ligament, in the lateral angle of the uterine cavity. Angular pregnancy can progress to term gestation but may be associated with obstetric complications such as rupture uterus, placental abruption, growth restriction, preterm delivery, retained placenta and postpartum hemorrhage [4]. ‘Cornual pregnancy’ is a conception that develops in the rudimentary horn of a uterus with a Mullerian anomaly [5].

Risk factors of interstitial pregnancy include previous ectopic pregnancy, ipsilateral or bilateral salpingectomy, in vitro fertilisation (IVF), or history of sexually transmitted disease [1]. In our case there was no known risk factor.

The ultrasonographic criteria for diagnosing interstitial pregnancy are: empty uterine cavity, gestational sac located eccentrically and 1 cm from the most lateral edge of uterine cavity, and a 5 mm myometrial layer surrounding the gestational sac [6]. ‘Interstitial line sign’ is also a useful criterion. It is the presence of an uninterrupted thin echogenic line between gestational sac and endometrium, suggesting that pregnancy is outside the uterine cavity [7]. 3D ultrasonography may also be used for accurate early diagnosis of interstitial pregnancy.

In 2003, Chan et al. reported 36 cases of interstitial ectopic pregnancies. 41.7% of interstitial ectopics were misdiagnosed at presentation. Most common misdiagnosis was intrauterine pregnancy, either viable or nonviable. Rupture of interstitial pregnancy occurred in 40% of these misdiagnosed cases which included two cases of rupture at an advanced gestation of 18-20 weeks [8].

Despite technological advancement in ultrasonography and improved diagnostic accuracy, interstitial ectopic may be misdiagnosed as intrauterine pregnancy due to lack of suspicion and expertise. Before presenting to us, our case was misdiagnosed as missed abortion and medical termination was attempted which could have proven to be catastrophic.

Interstitial ectopic pregnancies may be managed medically or surgically. Medical treatment with systemic methotrexate or local administration of methotrexate or potassium chloride into the ectopic gestational sac has been described for the treatment of interstitial pregnancy. Safety of methotrexate treatment of interstitial pregnancy depends on strict follow-up and the capacity to perform an emergency surgery if required. Close follow-up is a must, as 10-20% of patients will require either a second dose of methotrexate or surgery [1]. Success of medical management in interstitial pregnancy depends upon early diagnosis.

Surgical management offers definitive diagnosis and treatment. Location of pregnancy bulge in relation to round ligament is important in differentiating between angular and interstitial pregnancy. In angular pregnancy bulge is medial to round ligament whereas in interstitial pregnancy bulge is lateral to round ligament [5]. Previously, cornual wedge resection or hysterectomy by laparotomy were the main surgical options for management of interstitial ectopic pregnancy; however, the morbidity associated with these surgeries has led to a conservative surgery such as laparoscopic cornuostomy being preferred. Main steps in laparoscopic cornuostomy are incision over cornual bulge, removal of the products of conception, cornual repair and ensuring haemostasis [9]. Prior to cornual incision, various methods have been previously described to reduce intra-operative blood loss such as injecting vasopressin into peri-cornual area, electrocoagulating the incision area, endo-loop application as a para-cornual tourniquet and purse string suture around the cornual region [1]. In our case, we used a combination of two of the most simple and inexpensive techniques: vasopressin injection in the peri-cornual area and purse string suture around cornual region, which proved to be highly effective in reducing intraoperative blood loss.

While cornual wedge resection carries an increased risk of uterine rupture due to the loss of myometrium and extensive uterine scarring, cornuostomy removes the interstitial pregnancy, while preserving uterine myometrium reducing risk of uterine rupture [10]. Cornuostomy may also lead to lesser tubal damage than cornual wedge resection and may have better pregnancy outcomes [11].

In subsequent pregnancy, transvaginal ultrasound should be performed at five to six weeks to rule out recurrence and a planned caesarean delivery is the safest approach to avoid risk of uterine rupture in labour [12].

## Conclusions

Large interstitial ectopic pregnancy was correctly diagnosed by a combination of 2D and 3D ultrasonography after initial misdiagnosis and was successfully managed by laparoscopic cornuostomy; a conservative surgical approach with minimal blood loss. This case gives us the insight that clinicians should be aware of the potential of misdiagnosing an interstitial pregnancy as an intrauterine (angular) pregnancy. It also highlights that laparoscopic cornuostomy with simple and inexpensive techniques like intra-myometrial vasopressin injection and purse string suture can be highly effective in minimising intra-operative bleeding, reducing intra-operative time and morbidity.
